# Factors associated with physical activity in elderly nursing home residents: a path analysis

**DOI:** 10.1186/s12877-020-01676-8

**Published:** 2020-08-05

**Authors:** Jingxin Huang, Youqing Zou, Wentao Huang, Ye Zhou, Shanshan Lin, Jiaojiao Chen, Yutao Lan

**Affiliations:** 1 School of Nursing, Guangdong Pharmaceutical University, Guangzhou, China; 2 Faculty of Health, University of Technology, Sydney, Australia; 3 School of Cultures and Languages, University of Sydney, Sydney, Australia; 4 The First Affiliated Hospital of Guangdong Pharmaceutical University, Guangzhou, China

**Keywords:** Physical activity, Health belief model, Nursing home residents, Path analysis

## Abstract

**Background:**

Physical activity (PA) is low among elderly residents in nursing homes in China. We aimed to determine the factors that influence PA among elderly nursing home residents and their direct or indirect effects on PA levels.

**Methods:**

The PA levels of the participants were measured using the International Physical Activity Questionnaire, and their health beliefs were assessed using a self-developed 18-item questionnaire titled the ‘Health Beliefs of Nursing Home Residents Regarding Physical Activity’ in accordance with Health Belief Model (HBM) constructs. The correlations between HBM constructs and PA levels were analyzed and a regression-based path analysis was conducted to examine the relationships between HBM constructs and PA levels.

**Results:**

A total of 180 residents with a mean age of 82.5 years (standard deviation = 5.76) were recruited. Linear regression analysis revealed that self-efficacy (*p* < 0.001), perceived severity (*p* < 0.01), and cues to action (*p* < 0.01) were associated with the level of PA among nursing home residents. In the conceptual path model, self-efficacy, perceived severity, and cues to action had positive direct effects on the PA level, while perceived benefits and perceived barriers had indirect effects on the PA level.

**Conclusion:**

The residents’ self-efficacy, perceived severity, and cues to action were found to be important factors that can affect the design and implementation of educational programs for PA. A better understanding of such associations may help healthcare providers design informed educational interventions to increase PA levels among nursing home residents.

## Background

Physical inactivity was reported to be the fourth leading risk factor for non-communicable diseases. Poor PA leads to insufficient energy expenditure, which results in obesity and chronic diseases like type 2 diabetes and cardiovascular diseases [1]. PA is essential for healthy aging and offers many health benefits, including a reduced risk of chronic diseases [2] and premature death [3]. Moreover, the implementation of a PA program improves physical [4] and cognitive functions [5, 6], increases the quality of life, and decreases depressive symptoms [7] in elderly adults.

PA recommendations for elderly adults are a minimum of 150 min of moderate-intensity aerobic PA or at least 75 min of vigorous-intensity aerobic PA or an equivalent combination of both throughout the week [8]. The amount of PA is one of the determinants of the maintenance of independence in older adults [9]. However, elderly adults are more likely to be introverted, physically inactive, and reluctant to join social activities [7]. This situation is more evident in nursing home residents and is partly attributed to the limited space, fewer exercise coaches, and reduced physical function. Talbott et al. [10] reported that 51% of nursing home residents in the Czech Republic had a low PA level. In Australia, the daily average sitting time of older residents was 12.9 h [11]. In China, a previous study found that 68.2% of nursing home residents performed regular PA with light intensity [12]. Many nursing home residents in China live a sedentary lifestyle, and their main activities include reading, playing games, chatting, and watching television [13]. Both malnutrition and sarcopenia are common in nursing homes [14] and diminish the ability of older adults to perform PA and activities of daily living.

The most commonly used approach to describe PA is the Health Belief Model (HBM) in Physical Activity (HBMPA) [15]. Concepts from the HBM are widely used to explain individual psychological factors associated with PA.

HBM is one of the most important theoretical frameworks and has been shown to be useful in understanding and explaining preventive behaviors. As one of the most widely used psychological theories of health behavior, HBM is comprehensive for both psychological and environmental factors that influence health behavior and consists of six constructs: personal susceptibility and severity; efficacy; cues to action; physical, psychological, and financial barriers; benefits; and costs [16, 17]. According to the theory, a person’s readiness to adopt some health-promoting behaviors, including PA, depends on: a) a perception of vulnerability to health issues related to inactivity, b) a belief about the seriousness of possible consequences arising from the health conditions, c) a belief that suitable PA will effectively prevent the threat of negative health outcomes, and d) a belief that the anticipated benefits of the activity will outweigh the costs of action [18]. The theory provides a means to understand the attitude, behaviors, and educational needs of people. Regular PA or exercise can be best explained by HBM for individuals in both healthy and chronic states. A previous study [19] indicated that there is a positive correlation between perceived benefits and PA, but an inverse correlation between perceived barriers and PA. A strong sense of accomplishment and enjoyment and the enhancement of physical performance are positive benefits for older adults taking part in a new PA program [20]. Crombie et al. [21] identified pain (related to an existing condition), lack of interest, and facility accessibility as perceived barriers to daily activity for older adults. However, to date, few studies have assessed the PA level of elderly nursing home residents based on HBM constructs and associated factors. Self-efficacy, access to exercise resources, the perceived benefits of PA, and self-care ability positively impact the PA level of nursing home residents [12, 22], whereas limited space, fear of falling [23], and reduced physical or cognitive functioning [22] are obstacles to the maintenance of exercise behaviors.

Therefore, we aimed to examine the hypothesis that the health beliefs of nursing home residents regarding PA are associated with their PA level; specifically that those with more extensive health beliefs perform more PA and that PA can be associated with HBM constructs in elderly nursing home residents in a path model.

## Methods

### Study design

This is a cross-sectional study conducted from June 3rd to August 30th 2019 at Taikang Community (about 250 residents), Xiaocixuan Nursing Home (about 150 residents), and Shiqi Nursing Home (about 200 residents), which are nursing homes in three districts located to the east, west, and south of Guangzhou city, respectively. There are a lot of nursing homes in the city, but the above three were chosen because they had exercise facilities and offered onsite instructors, which enable a more comprehensive understanding and accurate identification of the associations between the influencing factors and the physical activity level of the residents. The institutional review board from the First Affiliated Hospital of Guangdong Pharmaceutical University approved the study. Participants were informed about the significance of the research and all aspects of the survey; written consent was obtained after confirming their willingness to participate.

### Participants

We included participants who 1) were aged 60 or above, 2) lived in the nursing home for over 3 months, 3) reported no difficulty in walking or were currently using a walking stick, and 4) showed no evidence of psychiatric conditions or difficulty in communicating. Those who were medically diagnosed with dementia or cognitive impairment, assessed using the Chinese version and norms of the mini-mental state examination (MMSE) [24], were excluded.

The sample size for performing a planned path analysis was estimated. A sample size of 5 to 10 per item (18 items in the instrument measuring the participants’ health beliefs) was needed to achieve a clear factor structure [25]. Therefore, the desired sample size was determined to be 90 to 180.

### Measures

Based on the domains of The Ecological Model of Active Living in a previous study [15], the contents of the questionnaire included the following: sociodemographic characteristics and health status, PA level, and the self-developed instrument named Health Beliefs of Nursing Home Residents Regarding Physical Activity (Table S1).

#### Sociodemographic characteristics and health status

Sociodemographic information including age, gender, marital status, education level, smoking status, alcohol consumption, weight, height, and diagnosis of chronic disease was collected. Self-reported medical conditions and duration of illness were confirmed by treatment and/or medication.

#### Physical activity assessment

The total PA level over the past 7 days was measured with one of the most widely used tools for assessing PA, namely the ‘International Physical Activity Questionnaire (IPAQ)’ [26]. The IPAQ was developed by researchers from several countries with the support of the World Health Organization and the US Centers for Disease Control and Prevention (www.ipaq.ki.se) and has great reliability and criterion validity, as confirmed in 12 countries [27]. Nonetheless, the reliability and criterion validity of this questionnaire among Chinese older adults were tested and confirmed [28]. The test-retest reliability coefficient between day 1 and day 9 was 0.84 (95% confidence interval, 0.80–0.87). Total PA measured with IPAQ-C moderately correlated with the pedometer-measured steps (Spearman and partial *r* = 0.33, *P* < 0.001) [28]. Liu and Hu reported that the test-retest reliability coefficient between day 8 and day 11 was 0.80 in a nursing home in China [13]. PA at different levels of intensity was assessed and any activity with a duration < 10 mins was eliminated. The total duration of PA was measured in minutes and converted to metabolic equivalent scores (MET mins·wk.^− 1^) for each type of activity. The MET score weights each type of activity by its energy expenditure, with 8 METs for vigorous activity, 4 METs for moderate activity, 3.3 METs for walking, and 1 MET for sitting. Total PA was categorized as low (< 600 MET mins·wk.^− 1^) and moderate to high (≥600 MET mins·wk.^− 1^), corresponding to less than 150 mins/wk. and more than 150 mins/wk. of moderate-intensity PA, respectively [29].

#### Health beliefs of nursing home residents regarding physical activity

The instrument was designed based on the HBM with a focus on the health beliefs of elderly nursing home residents regarding PA. Six major concepts of HBM (perceived susceptibility, perceived severity, perceived benefits, perceived barriers, cues to action, and self-efficacy) were adopted while designing the questionnaire. All parts of the HBM measurement were scored based on a 5-point Likert scale, ranging from 1 for ‘strongly disagree’ to 5 for ‘strongly agree’. The questionnaire originally comprised 21 items regarding the beliefs of elderly nursing home residents about PA. Participants either self-administered the questionnaire or received assistance from research assistants.

#### Validity and reliability of the health beliefs of nursing home residents regarding physical activity

Confirmatory factor analysis (CFA) was used to test the structural validity. A good model fit was defined as: χ^2^/df < 2, comparative fit index (CFI) > 0.90, and Root Mean Square Error of Approximation (RMSEA) < 0.06, while an acceptable model fit was defined as: χ^2^/df < 3, CFI = 0.80–0.89, and RMSEA< 0.10 [30]. The fit indices for the 18-item model were χ^2^/df = 2.287, CFI = 0.893, and RMSEA = 0.085, indicating an acceptable fit for the data [30].

The content validity was tested by an expert panel. Seven professionals from the fields of Health Education, Geriatric Nursing, Chronic Care, Rehabilitation Medicine and Kinesiology, and seven residents from the three nursing homes included consented to participate in the study as expert panel members. Both the Item Content Validity Index (I-CVI) and Scale Content Validity Index (S-CVI) were calculated. All I-CVI scores for the 18 items were higher than 0.78. The range of I-CVI was 0.786 to 1.0 and the S-CVI/ave. was 0.929, indicating good content validity [31].

The Cronbach’s alpha coefficient for each section, namely “perceived susceptibility”, “perceived severity”, “perceived benefits”, “perceived barriers”, “cues to actions”, and “self-efficacy”, was 0.790, 0.665, 0.751, 0.764, 0.696, and 0.865, respectively. The split-half reliability for each section was 0.791, 0.753, 0.603, 0.826, 0.655 and 0.877, respectively, which indicated an acceptable internal consistency [32].

### Data analysis

Descriptive statistics were presented as frequency (percentage) or mean (standard deviation) as appropriate. Continuous variables were analyzed by one-way analysis of variance (ANOVA) and the Student’s t-test, and categorical variables were analyzed by the chi-square or fisher’s exact tests as appropriate. Spearman correlation coefficient tests were conducted to test the associations between the HBM constructs, PA level, and sedentary time. Multiple linear regression analysis was used to examine the factors associated with the PA level. The results were considered statistically significant at *p* < 0.05. All of the above analyses were performed using IBM SPSS Statistics for Windows, version 21.0 (IBM Corp., Armonk, N.Y., USA).

#### Path analysis

To investigate the relationship between the PA level, health beliefs, and sociodemographic characteristics, a path analysis model was developed and tested using Amos 22.0 (Amos Development Corp, Meadville, PA, USA). The path analysis was used to explore the direct or indirect dependencies among a set of variables including the demographics and health belief model characteristics. The goodness of fit for the final model was assessed with the chi-square test and goodness of fit indices, such as the RMSEA, standardized root mean square residual (SRMR), goodness-of-fit index (GFI), adjusted goodness-of-fit index (AGFI), normed fit index (NFI), incremental fit index (IFI), Tacker-Lewis index (TLI), and CFI. The values for GFI, AGFI, NFI, IFI, TLI, and CFI range from 0 to 1, with values greater than 0.90 indicating a good fit. Conventionally, there is a good fit if the RMSEA and SRMR are less than 0.05.

## Results

Of the 213 nursing home residents we approached, 183 residents participated in the self-reported survey, leading to a response rate of 85.9%. Three participants were excluded due to incomplete information. The participants’ sociodemographic characteristics are described in Table 1. Among the 180 participants, the median age was 82.5 (range, 61–95). In our study, participants that were less educated, unmarried, or diabetic, and those with limited mobility tended to engage in a lower level of PA. The median PA was 25.7 mins/day (range, 0–180) and 884.5 MET mins·wk^-1^ (range, 0–6375). Physical inactivity was common among elderly adults in the nursing home, 50% of whom performed < 600 MET mins·wk^-1^ of PA and over 90.0% (163 participants) of whom reported low-intensity exercise such as walking. Participants engaging in > 600 MET mins·wk^-1^ of PA were more likely to be well educated (37.8%), have no chronic conditions, and score higher in the subscales for perceived severity and perceived benefits.

**Table 1:** Descriptive characteristics of participants stratified by physical activity (*N* = 180)

Characteristic		PA level (MET mins·wk^− 1^)			
	Total (N = 180)	< 600 (*N* = 90)	> = 600 (N = 90)	*F/t/χ*^2^	*P*
Age, Mean (SD), y	82.46 ± 5.76	82.93 ± 6.50	82.0 ± 4.90	1.101	0.272
Sex, n,%					
Men	60 (33.3)	26 (28.9)	34 (37.8)	−1.261	0.207
Woman	120 (66.7)	64 (71.1)	56 (62.2)		
Education level, n, %				−3.363	0.001
Illiterate	59 (32.8)	36 (40.0)	23 (25.6)		
Elementary school	40 (22.2)	23 (25.6)	17 (18.9)		
Junior High school	16 (8.9)	12 (13.3)	4 (4.4)		
Senior High school	17 (9.4)	5 (5.6)	12 (13.3)		
High Education	48 (26.7)	14 (15.6)	34 (37.8)		
Marital status, n, %				−2.174	0.030
Married	64 (35.6)	25 (27.8)	39 (43.3)		
Unmarried	116 (64.4)	65 (72.2)	51 (56.7)		
Height, Mean (SD), m	1.56 ± 0.09	1.54 ± 0.08	1.57 ± 0.98	−1.708	0.089
Weight, Mean (SD), kg	57.81 ± 9.29	57.60 ± 9.90	58.0 ± 8.73	−0.299	0.765
BMI, kg/m^2^				−0.439	0.661
< 18.5	3 (1.67)	2 (2.2)	1 (1.1)		
18.5–23.9	98 (54.4)	47 (52.2)	51 (56.7)		
24.0–27.9	65 (36.1)	33 (36.7)	32 (35.6)		
≥ 28.0	14 (7.8)	8 (8.9)	6 (6.7)		
Hypertension, n, %				3.733	0.053
Yes	124 (68.9)	68 (75.6)	56 (62.2)		
No	56 (31.1)	22 (24.4)	34 (37.8)		
CHD, n, %				0.378	0.539
Yes	68 (37.8)	32 (35.6)	36 (40.0)		
No	112 (62.2)	58 (64.4)	54 (60.0)		
Stroke, n, %				2.883	0.090
Yes	19 (10.6)	13 (14.4)	6 (6.7)		
No	161 (89.4)	77 (85.6)	84 (93.3)		
Diabetes, n, %				4.091	0.043
Yes	132 (73.3)	72 (80.0)	60 (66.7)		
No	48 (26.7)	18 (20.0)	30 (33.3)		
Cancer, n, %				0.000	1
Yes	2 (1.1)	1 (1.1)	1 (1.1)		
No	178 (98.9)	89 (98.9)	89 (98.9)		
COPD, n, %				0.000	1
Yes	1 (0.6)	1 (1.1)	0		
No	179 (99.4)	89 (98.9)	90 (100.0)		
Other diagnosed diseases, n, %				1.471	0.225
Yes	19 (10.6)	12 (13.3)	7 (7.8)		
No	161 (89.4)	78 (86.7)	83 (92.2)		
Limitation of mobility, n, %				8.229	0.004
Yes	40 (22.2)	28 (31.1)	12 (13.3)		
No	140 (77.8)	62 (68.9)	78 (86.7)		
Complication, n, %				0.000	1.000
Yes	8 (4.4)	4 (4.4)	4 (4.4)		
No	172 (95.6)	86 (95.6)	86 (95.6)		
Number of diagnosed diseases, Mean (SD)	1.58 ± 1.06	1.62 ± 1.09	1.54 ± 1.04	0.49	0.624
Duration of diagnosed disease, Mean (SD), years	12.7 ± 9.67	12.35 ± 8.88	13.04 ± 10.43	−0.483	0.629
Smoking status, n, %				0.424	0.515
Yes	10 (5.6)	6 (6.7)	4 (4.4)		
No	170 (94.4)	84 (93.3)	86 (95.6)		
Drinking status, n, %				0.719	0.396
Yes	26 (14.4)	11 (12.2)	15 (16.7)		
No	154 (85.6)	79 (87.8)	75 (83.3)		
Sedentary time, Mean (SD), minute	428.17 ± 153.06	524 ± 135.753	332.33 ± 100.58	10.762	0.001
Health Belief Model, Mean (SD)					
Perceived susceptibility	7.29 ± 2.15	6.74 ± 2.01	7.84 ± 2.16	−3.541	0.001
Perceived severity	8.35 ± 1.52	7.83 ± 1.53	8.85 ± 1.34	−4.766	0.001
Perceived benefits	8.24 ± 1.41	7.74 ± 1.42	8.73 ± 1.22	−5.038	0.001
Perceived barriers	6.29 ± 2.20	7.04 ± 1.77	5.53 ± 2.34	4.889	0.001
Cues to action	6.37 ± 1.90	5.7 ± 1.77	7.03 ± 1.80	−4.998	0.001
Self-efficacy	5.89 ± 2.52	4.36 ± 2.06	7.42 ± 1.96	−10.208	0.001

**Abbreviation:***PA* Physical activity, *BMI* Body mass index, *MET* Metabolic equivalent, *CHD* Coronary heart disease, *COPD* Chronic obstructive pulmonary disease

### Correlation analysis and multiple linear regression analyses

As shown in Table 2, we found statistically significant correlations between all of the HBM variables and PA levels (*p* all < 0.01). The range of the *r* coefficient was from 0.307 (for the relationship between perceived susceptibility and PA level) to 0.635 (for the relationship between self-efficacy and PA level), overall indicating weak (0.10–0.39) to moderate (0.40–0.69) correlations [33].

**Table 2:** Correlations between HBM constructs, physical activity level, and sedentary time (N = 180)

Item	Mean ± SD	PA level		Sedentary Time	
		*r*	*P*	*r*	*P*
Perceived susceptibility	7.29 ± 2.15	0.307	<0.01	−0.323	< 0.001
Perceived severity	8.35 ± 1.52	0.347	<0.01	−0.346	< 0.001
Perceived benefits	8.24 ± 1.41	0.387	<0.01	−0.414	< 0.001
Perceived barriers	6.29 ± 2.20	−0.470	<0.01	0.548	< 0.001
Cues to action	6.37 ± 1.90	0.387	<0.01	−0.403	< 0.001
Self-efficacy	5.89 ± 2.52	0.635	<0.01	−0.649	< 0.001

The multiple linear regression results are shown in Table 3. Only three of the HBM constructs (self-efficacy, perceived severity, and cues to action) were found to positively affect the PA level. Specifically, self-efficacy showed the strongest positive relationship with the total PA level.

**Table 3:** Multiple Regression Analysis for factors predicting physical activity level in elderly adults in nursing home

Variable	*B*	Standard error	*β*	*t*	*P*
Constant	− 391.542	73.805		−5.305	<0.001
Self-efficacy	50.075	5.306	0.549	9.438	<**0.001**
Perceived severity	25.972	8.921	0.172	2.911	<**0.01**
Cues to action	19.422	7.35	0.16	2.642	<**0.01**

### Path analysis

Based on the results of the linear regression model, we established a path analysis model to explore the relationship between the HBM constructs and daily PA. There were four hypotheses in the model: (i) ‘self-efficacy’ had a direct effect on total PA (0.52); (ii) ‘perceived severity’ had a direct effect on total PA (0.14); (iii) ‘cues to action’ had a direct effect on total PA (0.18); and (iv) ‘self-efficacy’ played a mediating role between ‘perceived benefits’ and ‘perceived barriers’ and total PA. In the present study, perceived severity referred to a poorer quality of life influenced by physical inactivity, including fatigue, limited social communication, and dependent lifestyle, leading to a heavy family burden.

**Fig. 1:**
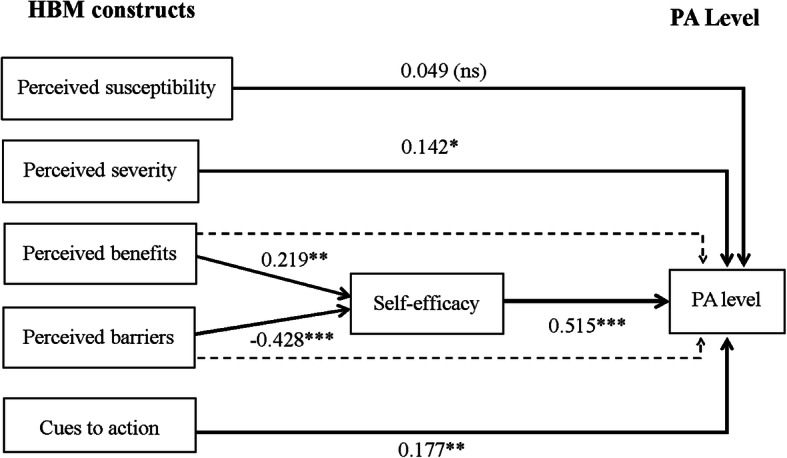
Path Model for Effects of HBM Constructs on PA level, Direct effects are represented by solid arrows; indirect effects are represented by dotted arrows. The β values are shown beside each arrow. *Significant at *p* < 0.05, **Significant at *p* < 0.01, ***Significant at *p* < 0.001; ns, not significant. Abbreviations: Health belief model (HBM), physical activity (PA)

As shown in Fig. 1, the fit indices were satisfactory for this model (χ^2^ = 5.664, χ^2^/df = 1.129, *p* = 0.342, RMSEA = 0.027, SRMR = 0.022, GFI = 0.991, AGFI = 0.950, CFI = 0.998, NFI = 0.986, IFI = 0.998, TLI = 0.993).

## Discussion

Our study found that half of the nursing home participants did not meet the current PA recommendations [29] as they performed PA less than 600 MET mins·wk.^− 1^, mostly with low intensity. This was consistent with the findings by Mary et al. [34] that lower intensity and less PA were common among older adults in China. We conducted path analyses and found three factors associated with PA. This is the first study that attempted to understand the factors associated with PA in a nursing home population in China.

We identified three variables (perceived severity, cues to action, and self-efficacy) in the path analysis that were the strongest predictors for the residents’ PA levels. The results suggested that variations in perceived severity, cues to action, and self-efficacy can be expected to alter PA engagement by about 57%. This was similar to the findings from Fatemeh [35] that the perceived severity of cardiovascular disease could affect PA behavior more than any other factor. Furthermore, we found that perceived barriers and benefits have indirect effects on the PA level, with self-efficacy serving as the mediating factor. A previous qualitative study based on the HBM framework [36] revealed that older adults performed PA for health and recreation, whereas emotional and social factors limited their confidence in behavior perception. In general, a behavior depends on self-efficacy, which is one of the clearest correlates with preventive health behaviors in adults and refers to an individual’s confidence in their ability to be physically active in particular situations [37]. Mullen & McAuley et al. [38] revealed that self-efficacy improved lower extremity function and functional performance and reduced functional limitations among older adults during PA. Specifically, for nursing home residents, increasing their walking frequency and duration promoted walking-related self-efficacy and their belief in their capability to adapt to their environment. The maintenance of healthy behaviors is one of the main challenges in health education and promotion. With higher confidence and the appropriate skills, individuals develop the self-efficacy to adhere to a planned exercise routine [39]. Additionally, the motivation for PA can be influenced by emphasizing the perceived benefits of PA such as healthy aging, positive health benefits for cardiovascular protection, physical function, and weight loss.

Regarding the implications for clinical practice, the overall aim of healthcare services for older adults living in a nursing home is to optimize their health, well-being, and quality of life to achieve the main goal of active living. Based on our findings, elderly residents in the nursing home were generally not inclined to adopt a sedentary lifestyle if they associated physically active behaviors with health. Moreover, proper educational programs for PA engagement interventions, adequate facilities, and personnel to provide exercise guidance for nursing home residents are needed. Previous studies conducted in China showed that vigorous PA, compared with low and moderate PA, was associated with a lower risk of stroke in elderly people [2]. Findings from Underwood et al. [40] also suggested that a moderate-intensity exercise program did not reduce depressive symptoms among residents of care homes. Increasing PA might be beneficial and contribute to various health benefits and decreased risks for diseases. However, due to the high prevalence of dementia among nursing home residents [6], preventive measures are necessary when implementing a PA program. Moreover, the optimal choice of a PA for nursing home residents is largely limited by specific risks, such as physical integrity and weakening [41].

Our study has some limitations. First, HBM constructs focus on a limited number of factors and ignore cultural, social, and economic factors and the previous experiences of elderly residents [42]. However, retrieving these sociodemographic factors from participants is difficult. In our study, we used a questionnaire to take into consideration several factors including educational background, marital status, smoking and drinking status, diagnosis of chronic diseases, and mobility capability. In addition, functional abilities that might significantly impact the PA level of the nursing homes’ residents were not measured directly, as all residents were assessed with the Barthel Index [43] when admitted to the nursing home. Instead, the resident’s chronic conditions and complications, which were closely related to their functional abilities, were surveyed. Second, although the self-report IPAQ was economical and convenient for PA level measurement, PA level might be overestimated among older adults due to recall bias. Third, our study was conducted with a relatively niche group of elderly individuals in a nursing home setting, which may limit its generalizability. However, this study will provide fundamental data on how to improve PA in this population of patients.

Findings from our investigation suggested that low-level PA was prevalent even in nursing homes with exercise facilities. Efforts should be made to develop exercise health education programs for older adults and empower them to engage in suitable daily exercises such as Tai Chi or active games in a safe environment. This will enable them to have positive experiences with exercise that improve their PA self-efficacy.

## Conclusions

In conclusion, we found that perceived severity, cues to action, and self-efficacy were associated with PA. HBM was found to be helpful for understanding the direct and indirect associations of cognitive determinants with the PA level among these individuals. This study provides evidence about factors that are useful for interventions to improve PA among elderly adults in nursing homes.

## Supplementary information

**Additional file 1 Table S1.** Health Belief of Nursing Home Residents Regarding Physical Activity.

## Data Availability

The datasets used and analyzed during the current study are available from the corresponding author on reasonable request.

## Abbreviations

PA: Physical activity
HBM: Health belief model
