# Supplementary material for: Factors associated with physical activity in elderly nursing home residents: a path analysis
Source: BMC Geriatr. 2020 Aug 5;20:274. doi: 10.1186/s12877-020-01676-8 (PMC7406386; doi:10.1186/s12877-020-01676-8)
Supplement: Supplementary file 1 — Additional file 1 Table S1. Health Belief of Nursing Home Residents Regarding Physical Activity. [file 12877_2020_1676_MOESM1_ESM.docx]

| **Table S1 Health Belief of Nursing Home Residents Regarding Physical Activity** | | | | | | | |
| --- | --- | --- | --- | --- | --- | --- | --- |
| 1. | Age | _______years old | | | | | |
| 2. | Gender | a) Man b) Woman | | | | | |
| 3. | Height | ________m | | | | | |
| 4. | Weight | ________kg | | | | | |
| 5. | Education level | a) Did not attend school b) Elementary school c) Junior High school d) Senior High school e) Higher Education | | | | | |
| 6. | Marital status | a) Married b) Unmarried c) Divorced d) Widowed | | | | | |
| 7. | Diagnosis of diseases | a) Hypertension b) Coronary heart disease c) Stroke d) Diabetes e) Cancer  f) Chronic obstructive pulmonary disease g) Other diagnosed diseases:________ | | | | | |
| 8. | Duration of the diagnosed disease | _______years | | | | | |
| 9. | Limitation of mobility | a) Yes b) No | | | | | |
| 10. | Complication | a) Yes b) No | | | | | |
| 11. | Smoking status | a) Yes b) No | | | | | |
| 12. | Drinking status | a) Yes b) No | | | | | |
| 13. | If I don't exercise, I am likely to suffer from chronic diseases such as diabetes, stroke, and coronary heart disease. | | Strongly  disagree | Somewhat disagree | Neither agree nor disagree | Somewhat agree | Strongly  agree |
| 14. | If I don't exercise, I am more likely to suffer from chronic diseases such as diabetes, stroke, and coronary heart disease than others. | | Strongly  disagree | Somewhat disagree | Neither agree nor disagree | Somewhat agree | Strongly  agree |
| 15. | I think that being physically inactive is a serious issue, which will affect my physical health. | | Strongly  disagree | Somewhat disagree | Neither agree nor disagree | Somewhat agree | Strongly  agree |
| 16. | I think that a decline in my health will affect my normal social activities and life. | | Strongly  disagree | Somewhat disagree | Neither agree nor disagree | Somewhat agree | Strongly  agree |
| 17. | I think that a decline in physical health will increase the family burden. | | Strongly  disagree | Somewhat disagree | Neither agree nor disagree | Somewhat agree | Strongly  agree |
| 18. | Daily physical activity helps maintain and improve my physical condition. | | Strongly  disagree | Somewhat disagree | Neither agree nor disagree | Somewhat agree | Strongly  agree |
| 19. | Doing physical activity every day makes me feel better overall. | | Strongly  disagree | Somewhat disagree | Neither agree nor disagree | Somewhat agree | Strongly  agree |
| 20. | Long-term, regular physical activity can prevent the occurrence of diabetes, stroke, coronary heart disease, and other diseases. | | Strongly  disagree | Somewhat disagree | Neither agree nor disagree | Somewhat agree | Strongly  agree |
| 21. | I think I may fall or get hurt while exercising. | | Strongly  disagree | Somewhat disagree | Neither agree nor disagree | Somewhat agree | Strongly  agree |
| 22. | There are no suitable sports fields and facilities around me. | | Strongly  disagree | Somewhat disagree | Neither agree nor disagree | Somewhat agree | Strongly  agree |
| 23. | I think doing physical activity is a waste of time. | | Strongly  disagree | Somewhat disagree | Neither agree nor disagree | Somewhat agree | Strongly  agree |
| 24. | There are no relevant professionals to guide me on what to do and how I exercise. | | Strongly  disagree | Somewhat disagree | Neither agree nor disagree | Somewhat agree | Strongly  agree |
| 25. | Doctors have advised me to increase my physical activity. | | Strongly  disagree | Somewhat disagree | Neither agree nor disagree | Somewhat agree | Strongly  agree |
| 26. | My family often reminds and encourages me to do more physical activity. | | Strongly  disagree | Somewhat disagree | Neither agree nor disagree | Somewhat agree | Strongly  agree |
| 27. | Mass media such as radio and television focuses on the benefits of physical activity. | | Strongly  disagree | Somewhat disagree | Neither agree nor disagree | Somewhat agree | Strongly  agree |
| 28. | I believe that I can continue to exercise. | | Strongly  disagree | Somewhat disagree | Neither agree nor disagree | Somewhat agree | Strongly  agree |
| 29. | Sometimes I encounter problems when doing physical activity, but I usually find a solution. | | Strongly  disagree | Somewhat disagree | Neither agree nor disagree | Somewhat agree | Strongly  agree |
| 30. | I think it is very easy to do physical activity. | | Strongly  disagree | Somewhat disagree | Neither agree nor disagree | Somewhat agree | Strongly  agree |

**Sub-dimensions for Scoring:**

Perceived susceptibility: Items 13 and 14

Perceived severity: Items 15, 16 and 17

Perceived benefits: Items 18, 19 and 20

Perceived barriers: Items 21, 22, 23 and 24

Cues to action: Items 25, 26 and 27

Self-efficacy: Items 28, 29 and 30
